# Pulmonary vein stenosis detection by early cardiac magnetic resonance imaging post-atrial fibrillation ablation

**DOI:** 10.1186/1532-429X-14-S1-P208

**Published:** 2012-02-01

**Authors:** Lowell Chang, Divya Ratan Verma, Eugene Kholmovski, Sathya Vijayakumar, Nathan S Burgon, Paul A Anderson, Nassir F Marrouche, Christopher McGann

**Affiliations:** 1 Cardiology, The University of Utah School of Medicine, Salt Lake City, UT, USA

## Summary

Identification of early post-atrial ablation MRI characteristics can help predict the development of significant chronic pulmonary stenosis.

## Background

Pulmonary vein stenosis (PVS) is a rare complication of atrial fibrillation (AF) ablation with a rate of significant stenosis, defined as >50%, of 1.3% in a recent multicenter study. As significant morbidity is associated with PVS, detection and surveillance with non-invasive imaging is routine. In this study, we hypothesized that prediction of PVS can be determined with early MRI to identify patients at risk for this complication.

## Methods

**Figure 1:**
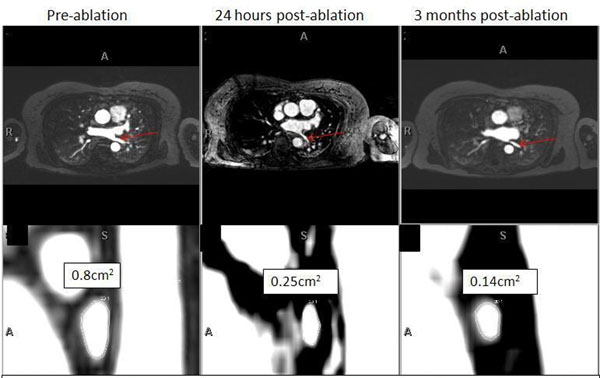
Example of a patient who developed left inferior pulmonary vein (LPV) narrowing post-ablation. **Top row**. Serial axial MRA images showing normal LIPV per-ablation with progressive narrowing 24 hours post-ablation and 3 months post-ablation (red arrows). **Bottom row**: Corresponding ostial/proximal LIPV cross-sectional views and area measurements.

A single-center, retrospective, 1:1 cohort to control matched study including patients with (23) and without (23) significant PVS 3 months post-ablation (3moPA) was performed. Study groups were selected from 925 patients who underwent AF ablation and serial MRI scanning. Inclusion criteria for both groups required a full set of three MRI scans: pre-ablation (pre), 24 hours post-ablation (24hrPA), and 3moPA. MRI scanning was performed on a 1.5T or 3T Siemens magnet. Of the 925 patients, 28 were found with significant stenosis on MRA 3moPA. The final PVS study cohort included 23/28 patients as 5 were excluded secondary to incomplete MRI scan sets. The control group was comprised of 23 age and sex-matched patients without significant stenosis 3moPA. PV cross-sectional areas were measured at the ostial/proximal portion of the vessels by 3D MRA (Figure 1). In patients with multiple stenotic PV’s, the most stenotic vein was selected for analysis.

## Results

**Figure 2:**
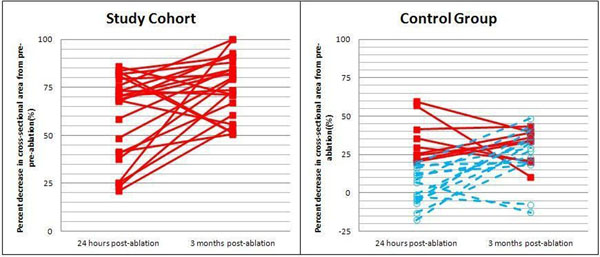
Percent decrease in pulmonary vein cross-sectional areas from pre-ablation at 24 hours post-ablation and 3 months post-ablation. Side-by-side comparison of study cohort to control group. Red solid lines representing patients with >20% narrowing at 24 hours post-ablation. Blue dashed lines representing patients with ≤20% narrowing at 24 hours post-ablation. **Note**: all of 23 patients in the study cohort had >20% pulmonary vein narrowing as compared to 9 of 23 patients in the control group at 24 hours post-ablation.

Out of 925 patients, we found 3% (28/925) incidence of significant PVS 3moPA. Of the 23/28 patients in the PVS study cohort, early PV narrowings of >20% on the 24hrPA scan were found in all (23/23, 100%) compared with significantly fewer in the control group (9/23, 39%) (p < 0.001) (Figure 2). Maximal stenosis found 3moPA involved the left inferior pulmonary vein (LIPV) in 74% (17/23) of the patients and 26% (6/23) in the other veins. Average baseline pre-ablation PV cross-sectional area for the stenotic veins in the study cohort was significantly smaller, 1.19 ± 0.61 cm2, compared to the control group, 2.01 ± 0.73 cm2 [p<0.001, 95% CI -1.22 to -0.42].

## Conclusions

All patients with significant PVS 3moPA demonstrated signs of pulmonary vein narrowing (>20%) on the 24hrPA MRI scan. In addition, small PV caliber on baseline imaging appears to predispose to PVS with the LIPV involved disproportionately. Overall, MRI image characteristics are useful for predicting PVS and may help determine which patients will benefit from surveillance imaging.

## Funding

N/A

